# Prevalence of cystic echinococcosis among livestock in pastoral and agro-pastoral areas in Uganda

**DOI:** 10.1017/S0031182023001154

**Published:** 2024-01

**Authors:** Leonard Omadang, Martin Chamai, Francis Ejobi, Joseph Erume, Peter Oba, Michael Ocaido

**Affiliations:** 1 College of Veterinary Medicine, Animal Resources and Biosecurity, Makerere University, Kampala, Uganda; 2 Faculty of Agriculture and Animal Sciences, Department of Animal Production and Management, Busitema University, Arapai Campus, Soroti, Uganda; 3 Directorate of Research and Innovations, Faculty of Health Sciences, Soroti University, Soroti, Uganda; 4 National Agricultural Research Organization (NARO), Abi Zonal Agricultural Research and Development Institute (Abi ZARDI), Arua, Uganda

**Keywords:** agro-pastoral, cystic echinococcus, livestock, organs, pastoral, prevalence, Uganda

## Abstract

Cystic echinococcosis (CE) remains a significant challenge in Uganda with precise status largely undocumented in most communities. To determine CE prevalence, post-mortem examination was done on 14 937 livestock (5873 goats, 1377 sheep, 3726 zebu cattle and 3054 Ankole cattle) slaughtered in abattoirs in the districts of Moroto in Karamoja region, Kumi in Teso region and Nakasongola and Luwero in Buganda region. The overall CE prevalence was 21.9% in sheep, 15.2% in zebu cattle, 5.5% in goats and 2.1% in Ankole cattle. Moroto district had a higher prevalence of CE than other districts with 31.3% in zebu cattle, sheep 28%, goats 29.1% and (0%) in Ankole cattle. On organ locations, the lungs were the most affected in all livestock in all the study areas. Considering cyst fertility, 33.9, 1.7 and 6.4% of Ankole cattle, sheep and zebu cattle respectively had fertile cysts in the liver while 4.5% of goats and 4% Ankole cattle had fertile cysts in the lungs. In conclusion, CE is widespread and occurs among cattle, sheep and goats in pastoral and agro-pastoral areas in Uganda. Therefore, there is an urgent need to create awareness among the communities on role of livestock in CE epidemiology and transmission.

## Graphical abstract

**Figure:**
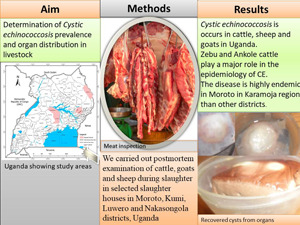

## Introduction

Cystic echinococcosis (CE) is a cosmopolitan often under-reported neglected disease of economic and public health significance (McManus *et al*., 2003; Nakao *et al*., 2007; Battelli, 2009; Da Silva, 2010). Although humans are affected as accidental hosts, CE affects both wild and domestic animals. It is caused by the metacestodes of parasitic tapeworms belonging to the family Taeniidae and genus *Echinococcus* (Spickler, 2020). Extensive research has shown transmission to livestock (intermediate hosts) and humans (accidental hosts) is through the consumption of *Echinococcus* spp. eggs, from fecal matter of infected, unrestricted, freely roaming and in most cases un-wormed domestic and wild canids (definitive hosts), present in contaminated pastures, water, vegetables and fresh fruits (Torgerson and Budke, 2003; Eckert and Deplazes, 2004). Canids harbour mature adult worms in their intestines, producing eggs that, once ingested by the intermediate hosts, hatch into larvae, enter the circulation and slowly develop into fluid-filled structures lodged as cysts in various host organs, mainly in lungs and liver, thus compromising their functions (Eckert and Deplazes, 2004).

Hydatidosis is mainly caused by *Echinococcus granulosus* sensu lato complex comprising the following strains: *E. granulosus* sensu stricto (G1, G2 and G3), *Echinococcus canadensis* incorporating G6-camel strain, pig strain (G7), G9 up to G8 and G10 (the cervid strains), *Echinococcus equinus* (G4), *Echinococcus ortleppi* (G5) and *Echinococcus felidis* – the lion strain (Hüttner *et al*., 2008; Alvarez Rojas *et al.*, 2014; Romig *et al.*, 2015; Shariatzadeh *et al*., 2015; Thompson, 2017).

CE causes considerable losses of productivity in livestock measured in terms of weight loss, lowered meat quality and birth rates, coupled with economic losses as a consequence of organ condemnations during post-mortem (PM) meat inspections (Craig *et al*., 2007; Cardona and Carmena, 2013). Studies in ruminants in Iran revealed total liver and lung condemnations of 36.08 and 48.04% respectively, estimated at US$ 459 659.6 (Ahmadi and Meshkehkar, 2011); and in Turkey, US$ 89.2 million (72.2–107.9) (Sariözkan and Yalçin, 2009); in Australia, ranged from AU$ 36 683 in 2016 to AU$ 163 006 in 2014 in beef cattle (Wilson *et al*., 2020); in Ethiopia, in bovines: 410 755.90 Ethiopian Birr (ETB; 30 202.64 US$; 1 US$ = 13.60 ETB) (Bekele and Butako, 2011); 25 608 ETB (Kebede *et al*., 2009*b*) and 1 691 266 200 ETB (101 203 734 US$) (Fromsa and Jobre, 2012). In recent years, there has been an increasing interest in CE research worldwide.

*Echinococcus granulosus* is known to occur in over 100 countries globally (Otero-Abad and Torgerson, 2013; Deplazes *et al*., 2017); a high prevalence was recorded in the Mediterranean countries (Bosco *et al*., 2021), Pakistan (Khan *et al*., 2018), Turkey (Sariözkan and Yalçin, 2009), Saudi Arabia (Almalki *et al.*, 2017), Australia (Lightowlers *et al*., 2021), China (Nakao *et al*., 2010; Fu *et al*., 2021) and South America (Larrieu *et al*., 2001).

In Africa, it has been found to be highly prevalent among domestic and wild animals (Ohiolei *et al*., 2020). So far, limited studies in Uganda have revealed a CE prevalence of 1.84% in humans (Othieno *et al*., 2016); 12.2 and 66.4% in dogs (Inangolet *et al*., 2010; Oba *et al*., 2016), identification of *E. granulosus sensu* stricto G1 sheep strain and *E. canadensis* G6/7 camel strain in livestock in Karamoja (Chamai *et al*., 2016). Also *E. felidis* was found in lions and warthogs (Hüttner *et al*., 2008). Hence in Uganda, at least 4 strains of *E. granulosus* sensu lato complex do occur. Also, earlier studies had been conducted on peoples’ attitudes and practices towards CE persistence in livestock among pastoral and agro-pastoral areas in Uganda (Oba *et al*., 2016; Omadang *et al*., 2018). Furthermore, such studies had been conducted in Kenya (Njoroge *et al*., 2002; Addy *et al*., 2012; Kagendo *et al*., 2014; Mbaya *et al*., 2014; Odongo *et al*., 2018; Nungari *et al*., 2019), Ethiopia (Sissay *et al.*, 2008; Kebede *et al*., 2011; Terefe *et al*., 2012; Kumsa and Mohammedzein, 2014; Tigre *et al*., 2016; Serda and Jago, 2017), Sudan (Omer *et al*., 2010) and Tanzania (Ernest *et al*., 2009; Miran *et al.*, 2017).

However, much less is known about prevalence of CE in livestock in Uganda. To address this gap, this study was undertaken to establish the prevalence of CE among slaughtered livestock in pastoral and agro-pastoral areas in Uganda. These studies are important in informing policy about the urgency needed to control CE among these rural livestock-keeping communities in Uganda.

## Materials and methods

**Figure 1.**
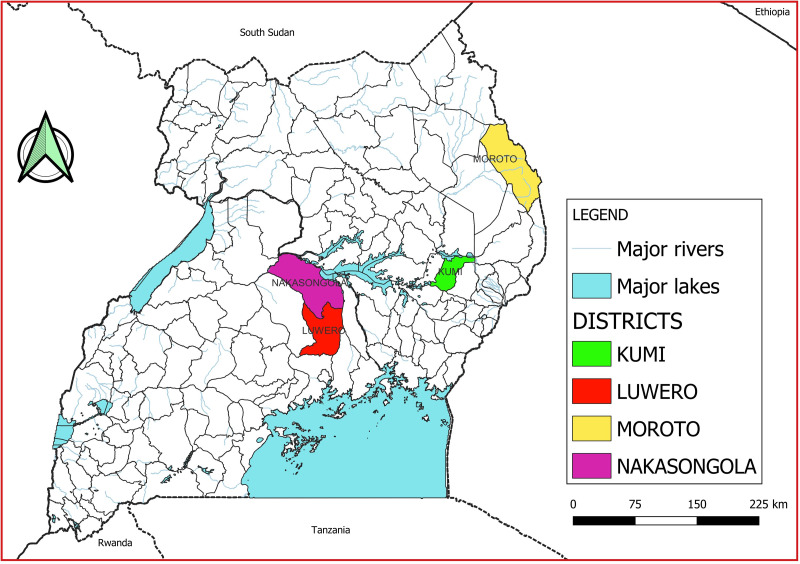
Map of Uganda showing study areas.

The study was carried out from March 2019 to February 2020 in selected designated slaughter abattoirs in the districts of Moroto in Karamoja region, Kumi in Teso region and Luwero and Nakasongola in Buganda region, Uganda (see Fig. 1). Moroto district was selected to represent pastoral areas (keeping livestock) in Karamoja region, Kumi to represent agro-pastoral (practice of both livestock keeping and crop agriculture) areas in eastern Uganda and Luwero and Nakasongola districts to represent agro-pastoral areas in central Uganda

A stratified random sampling procedure was used during this study. A total of 14 937 livestock were inspected. Details are shown in Table 1.

**Table 1.** Number of livestock examined in different districts

Species	District				Over all
	Moroto	Kumi	Luwero	Nakasongola	
Goat	1012	1406	2647	808	5873
Sheep	1049	328	0	0	1377
Zebu cattle	1698	1590	435	3	3726
Ankole cattle	0	0	2849	1105	3954
Exotic cattle	0	0	7	0	7
Total	3759	3324	5938	1916	14 937

PM examination was undertaken on slaughtered cattle, sheep and goats. Most livestock brought for slaughter were local indigenous breeds from the neighbouring local livestock markets. Animals for PM inspection were randomly selected before entry to the slaughter facility. Selected animals were marked with paint for easy traceability. For each selected animal, information on species, breed, sex and origin/market purchased from, date and age were recorded. The animals were grouped based on teeth eruption (Torell *et al.*, 2003; Eubanks, 2012; Whiting *et al*., 2013) as ‘young’ when aged below 3 years and as ‘adults’ when aged above 3 years.

During PM inspection, each organ was visually assessed, palpated and incised to detect embedded hydatid cysts (Soulsby, 1982). Recovered cysts were collected in air-tight labelled containers. The nature of the examinations performed would also detect other tapeworm parasites and these would also be recorded. For each positive organ, all recovered cysts were counted. Cyst diameters were measured to determine their sizes: ‘small’ when measuring below 4 cm, ‘medium’ measuring 4–8 cm and ‘large’ measuring beyond 8 cm, as described by Kebede *et al.* (2009*a*). Recovered cysts were further characterized to determine their fertility and sterility status using standard protocols developed by Soulsby (1982). The cyst wall was carefully separated from host tissues, incised with a sterile scalpel blade and the contents were poured into a clean Petri dish and examined under a microscope under ×40 magnification. The presence of protoscolices seen as white dots on the germinal epithelium were categorized as ‘fertile’ while those without as ‘infertile’. Cyst infertility was further classified as ‘sterile’ when having turbid contents, as ‘calcified’ when it was gritty when cut and as ‘degenerated’ when it had pus.

The nature of the examination performed would also detect other tapeworm parasites notably *Cysticercus tenuicollis* cysts and these would also be recorded. This is because *Taenia hydatigena*, a tapeworm responsible for *C. tenuicollis* infection in sheep and goats shares a strong association with *Cysticercus* and *Echinococcus* in terms of the definitive hosts (dog) and intermediate hosts (herbivores) and therefore have the same risk factors and infection levels (Eckert *et al.*, 1984; Murell, 2005).

### Data analysis

Data were entered into an MS Excel 16.0 spreadsheet. Statistical analysis was performed using R Statistical software program (R Core Team, 2022). Chi-squared tests and odds ratios (ORs) at 95% confidence interval (CI) were used to examine differences in CE prevalence by livestock species, age, sex, districts and organs affected. Values of *P* < 0.05 were considered statistically significant.

## Results

The overall prevalence of CE in livestock by sex and age showed that infection in males (6.2%) was higher than those in females (2.3%), and ‘older’ animals >3 years were also more infected (8.2%) than ‘young’ animals (0.3%) as shown in Table 2. The prevalence of CE among livestock species by district is shown in Table 3. A very highly significant CE prevalence of 29.1% was found in goats in Moroto (*χ*^2^ = 4473.7, *P* < 0.001) than in Kumi (0.6%), Luwero (0.6%) and Nakasongola (0.2%); similar trends were also observed in sheep (28%) in Moroto district (*χ*^2^ = 981.4, *P* < 0.001), Kumi district (2.1%) and none (0%) in both Luwero and Nakasongola districts; and in zebu cattle (31.3%; *χ*^2^ = 631.3, *P* < 0.001) in Moroto, Kumi (1.8%) and Luwero districts (1.6%) and none in Nakasongola district. CE prevalence was significantly higher (*P* < 0.05, *χ*^2^ = 4.04) in Ankole cattle (2.8%) in Nakasongola district than in Luwero (1.8%) and none (0%) in both Kumi and Moroto districts. No exotic cattle examined only in Luwero district was found with CE cysts. No sheep were slaughtered in Luwero and Nakasongola districts. Similarly, no Ankole cattle were slaughtered in Moroto and Kumi districts.

**Table 2.** Overall prevalence in all regions by sex and age

Variable	Factor	Prevalence (*n* = 14 937)	*χ*^2^ statistic	OR (95% CI)	*P* value
Age (years)	<3	40 (0.3%)	0.46166	1.12 (0.81, 1.60)	0.5751
	>3	1231(8.2%)			
Sex	Female	349(2.3%)	25.082	1.39 (1.22, 1.58)	0.00000042
	Male	922(6.2%)

**Table 3.** Percentage CE prevalence with 95% CIs among livestock species by district

Livestock species	District				Overall
	Moroto	Kumi	Luwero	Nakasongola	
Goats	29.1 (26.3–31.9)	0.6 (0.2–1.0)	0.6 (0.3–0.9)	0.2 (0–0.5)	5.5 (4.9–6.1)
Sheep	28 (25.3–30.7)	2.1 (0.5–3.7)	–	–	21.9 (19.7–24.1)
Zebu cattle	31.3 (29.1–33.5)	1.8 (1.1–2.5)	1.6 (0.4–2.8)	–	15.2 (14.0–16.4)
Ankole cattle	–	–	1.8 (1.3–2.3)	2.8 (1.8–3.8)	2.1 (1.7–2.5)

Regarding percentages of CE prevalence by organ infected among livestock species per district, in Moroto, the lungs of 27.7% of goat, 24% of sheep and 20.3% of zebu cattle had cysts significantly higher than that of goats (0.6%), sheep (2.1%) and zebu cattle (0.8%) in Kumi; in Luwero district, lungs of 0.9% of zebu cattle, 1.1% of Ankole cattle and 0.5% of goats and, in Nakasongola district, 1.6% of lungs of Ankole cattle and 0.2% of goats. Cyst infection in the livers was not reported in goats, sheep and zebu cattle in Nakasongola district. In Moroto district, the livers of zebu cattle (12.8%), sheep (5.3%) and goats (3.6%) were more infected than those of the same animals in Kumi and Luwero districts, and none in goats, sheep and zebu cattle in Nakasongola district and in sheep in Luwero district. Ankole cattle had almost similar prevalence in lungs in Luwero (1.1%) and Nakasongola (1.6%) compared to 0.8 and 1.6% in the liver respectively, and no cysts were recovered from Ankole cattle in Kumi and Moroto districts as shown in Table 4.

**Table 4.** Percentage organ CE prevalence (number positive in brackets) by species across all districts

Livestock species	Organ	District				Overall
		Moroto	Kumi	Luwero	Nakasongola	
Goats	Lung	27.7 (280)	0.6 (8)	0.5 (12)	0.2 (2)	5.1 (302)
	Liver	3.6 (36)	0.1 (1)	0.2 (5)	0	0.7 (42)
	Lung and liver	2.1 (21)	0	0.04 (1)	0	0.37 (22)
	Lung, liver and spleen	0.1 (1)	0	0	0	0.02 (1)
Sheep	Lung	24 (252)	2.1 (7)	0	0	18.8 (259)
	Liver	5.3 (56)	0	0	0	4.1 (56)
	Lung and liver	1.2 (13)	0	0	0	0.9 (13)
	Lung, liver and spleen	0.3 (1)	0	0	0	0.1 (1)
Zebu cattle	Lung	20.3 (345)	0.9 (15)	0.9 (4)	0	9.8 (364)
	Liver	12.8 (218)	1.2 (19)	0.9 (4)	0	6.5 (241)
	Lung and liver	1.8 (31)	0.3 (5)	0.2 (1)	0	1.0 (37)
	Lung, liver and spleen	0.06 (1)	0	0	0	0.03 (1)
Ankole cattle	Lung	0	0	1.1 (32)	1.6 (18)	1.3 (50)
	Liver	0	0	0.8 (23)	1.6 (18)	1.0 (41)
	Lung and liver	0	0	0.04 (1)	0.0	0.03 (1)
	Lung, liver and spleen	0	0	0.1 (3)	0	0.08 (3)

Lung prevalence in goats was 100% in Nakasongola district, 88.9% in Kumi, 88.3% in Moroto and 70.6% in Luwero; in sheep was 100% in Kumi and 81.6% in Moroto; in zebu cattle was 50% in Luwero, 44.1% in Kumi and 61.1% in Moroto; only in Nakasongola 55.2% of the lungs of Ankole cattle were affected.

In the liver: in zebu cattle, 55.9% in Kumi and 38.7% in Moroto were infected, 39.7% and 50% in Ankole cattle in Luwero and Nakasongola districts were affected; and in goats, 29.4% in Luwero, 11.4% in Moroto and 11.1% in Kumi districts were affected. Details are shown in Table 5. Among cyst sizes, small cysts (72.1%) formed the bulk of infection in the lungs than in the liver (53.3%); mixed organ infections had more medium-sized infection (44.4%) than the liver (23.3%) and lungs (18.6%); the large cysts formed in the liver (23.3%) and (9.2%) in the lungs, as shown in Table 6. The number of cysts recovered per CE-infected organ is shown in Table 7. The lungs harboured the highest number of single and multiple cysts (890) compared to the liver (296). The mean cyst counts was 1.4 ± 0.03 per CE-infected liver, 1.4 ± 0.9 per infected lung, 3.2 ± 0.23 per mixed lung and liver infection and 5 per mixed lung, liver and spleen infection.

**Table 5.** Percentage composition of certain organs being CE-infected compared to other CE-infected organs per livestock species per district

Species		Moroto	Kumi	Luwero	Nakasongola
Goats	Lung	88.3	88.9	70.6	100
	Liver	11.4	11.1	29.4	0
	Spleen	0.3	0	0	0
Sheep	Lung	81.6	100	0	0
	Liver	18.1	0	0	0
	Spleen	0.3	0	0	0
Zebu cattle	Lung	61.1	44.1	50	0
	Liver	38.7	55.9	50	0
	Spleen	0.2	0	0	0
Ankole cattle	Lung	0	0	55.2	50
	Liver	0	0	39.7	50
	Spleen	0	0	5.2	0

**Table 6.** Percentage of organs of livestock examined with small-, medium- and large-sized cysts

Organ	Small	Medium	Large
Lung (*n* = 944)	72.1	18.6	9.2
Liver (*n* = 317)	53.3	23.3	23.3
Lung and liver (*n* = 132)	47.7	31.1	21.2
Lung, liver and spleen (*n* = 9)	33.3	44.4	22.2

**Table 7.** Percentage number of cysts recovered per CE-infected organ recovered

Number of cysts recovered	Number of organs			
	Lung	Liver	Lung and liver	Lung, liver and spleen
1	667	223	1	0
2	145	49	33	0
3	60	15	26	1
4	18	8	12	1
5	0	0	0	1
6	0	1	3	1
7	0	0	3	1
8	0	0	3	0
Total	890	296	81	5

Percentage composition of status of CE cysts recovered from different organs per livestock species examined is shown in Table 8. Ankole cattle (33.4%), zebu cattle (6.4%) and sheep (1.7%) had fertile cysts in the liver while only goats had fertile cysts in the lungs (4.5%).

**Table 8.** Percentage composition of status of CE cysts recovered from different organs per livestock examined

Species	Organ	Calcified	Sterile	Degenerating	Fertile
Goat	Lung (*n* = 334)	89.5	1.2	4.8	4.5
	Liver (*n* = 40)	82.5	2.5	15	0
Sheep	Lung (*n* = 263)	97	1.1	1.9	0
	Liver (*n* = 58)	91.4	0	6.9	1.7
Zebu cattle	Lung (*n* = 393)	34.6	58	7.4	0
	Liver (*n* = 264)	23.5	58	12.1	6.4
Ankole cattle	Lung (*n* = 50)	34	40	22	4
	Liver (*n* = 59)	13.6	37.3	15.3	33.9

Moreover, we were not interested in other parasites in this study, the proximity and similarity of this tapeworm (*C. tenuicollis*) cysts made them obvious as present within the protocol/procedure followed for examination. The prevalence of *C. tenuicollis* cysts therefore was found to be high in both goats and sheep. Details are as shown in Table 9.

**Table 9.** Percentage prevalence (number positive in brackets) of *Cysiticercus tenuicollis* in goats and sheep per district

Species	Moroto	Kumi	Luwero	Nakasongola	Overall
Goat	40 (405)	36.4 (512)	33.4 (883)	34.8 (281)	35.4 (2081)
Sheep	24.6 (258)	19.2 (63)	–	–	23.3 (321)

## Discussion

The communities in the study areas were pastoralists in Moroto district in Karamoja region; agro-pastoralists in Kumi district in Teso region and Luwero and Nakasongola districts in Buganda region. The overall CE prevalence among livestock examined was found to be 21.9% in sheep, 15.2% in zebu cattle, 5.5% in goats and 2.1% in Ankole cattle. Moroto district was found to have higher prevalence of CE than other districts; cattle had CE prevalence of 31.3%, sheep 28% and goats 29.1%. This could be attributed to differences in life styles affecting the level of dog–livestock interactions, sources and level of water availability. This may be because livestock keepers in Moroto were practicing transhumance thus seasonally move during periods of severe water shortages (dry season) to areas with water and pasture. This led to high concentration of livestock with the accompanying dog population, kept for security purposes, increasing the potential of transmission of CE between livestock, humans and dogs. In such a situation, dogs’ fecal matter easily contaminates water sources with *E. granulosus* eggs. However in agro-pastoral areas (practicing both crop and livestock farming) in Teso, livestock farmers lived a sedentary life style – access to clean water was relatively better, water sources were often bore holes and protected wells where dog access was limited or completely denied. In agro-pastoral areas in Nakasongola and Luwero farmers’ practiced agro-pastoralism – sources of water were open unprotected pools, dams, springs, lakes, rivers and swamps. These water sources were being shared among humans, livestock and dogs. In these areas, there is loose association of dogs with livestock keepers because dogs are mostly owned by communities for hunting (Oba *et al*., 2016).

CE prevalence found in cattle in Moroto was similar to that reported in Maasailand in Kenya (25.5%) by Addy *et al*. (2012) and in Ethiopia (28%) by Debas and Ibrahim (2013). CE prevalence found was lower than that reported in Ethiopia by Serda and Jago (2017) and Mandefro *et al*. (2019) of 52.5 and 54%, respectively, in Adama municipal abattoir, south-eastern Ethiopia, 34.5% in Bahir Dar, North West Ethiopia (Kebede *et al.*, 2009*a*), 40.5% in Addis Ababa Abattoirs Enterprises (Terefe *et al*., 2012), 46.8% central Oromia, Ethiopia (Getaw *et al*., 2010) and 48.7% in Arusha, Tanzania (Ernest *et al*., 2009). CE found in Moroto was higher than that reported in Kajiado, Kenya: of 14.3% (Nungari *et al*., 2019) and 5.3% in Migori county (Kere *et al*., 2019); in Ethiopia: of 21% in Addis Ababa Abattoir Enterprises (Kumsa, 2019), in Gessa (17%) and Wolaita Sodo (16.85%), southern Ethiopia (Bekele and Butako, 2011; Mesfin *et al*., 2022) and 15.2% in north-western Ethiopia (Kebede *et al*., 2011). Despite being in the same ecological zones of ‘horn of Africa’, these differences in prevalence could have been due to variances in environmental conditions, livestock population densities, livestock migrations and grazing systems (Njoroge *et al*., 2002; Serda and Jago, 2017), diverse cultures and *E. granulosus* strains (McManus, 2006) and awareness (Omadang *et al*., 2018) in these areas.

CE prevalence of 28% in sheep found in Moroto was similar to 29.5% in sheep reported among sheep slaughtered in restaurants in south-western Oromo (Kumsa and Mohammedzein, 2014) and 29.3% in central Oromia, Ethiopia (Getaw *et al*., 2010). CE prevalence of sheep in Moroto was much lower than that reported earlier in CE hyperendemic zones in southern Italy of 62.9% (Bosco *et al*., 2021) and in eastern Ethiopia of 68% (Sissay *et al.*, 2008). Lower CE prevalence rates of sheep had also been reported in Kenya of 14.9% by Nungari *et al*. (2019) in Kajiado, 16% by Odongo *et al*. (2018) in Narok county and 0.1% by Kere *et al*. (2019) in Migori county; and 17.1% in Ethiopia (Tadesse *et al*., 2014).

CE prevalence of 29.1% found in goats was similar to that reported in CE hyperendemic zones in southern Italy of 28% (Bosco *et al*., 2021), 24.8% in south-western Oromo, Ethiopia (Kumsa and Mohammedzein, 2014) and 22.2% in Ngorongoro, Tanzania (Miran *et al.*, 2017). Much higher CE prevalence of 65% in goats had been reported (Sissay *et al.*, 2008) in south-eastern Ethiopia and 63.8% in Tanzania (Ernest *et al*., 2009). However, lower goat CE prevalence rates had also been reported in Kenya of 16% (Odongo *et al*., 2018) and 14.8% (Nungari *et al*., 2019).

An earlier study (Othieno *et al*., 2016) also showed high prevalence of CE in humans using ultrasound screening. The risk factors were found to be, owning of a large number of livestock, large dog populations found in livestock grazing and watering areas, home livestock slaughters and feeding of dogs with cystic offals (Oba *et al*., 2016; Othieno *et al*., 2017; Omadang *et al*., 2018). Overall, these were promoted by limited awareness within the community of the epidemiology of CE (Omadang *et al*., 2018; Othieno *et al*., 2018). The observation shows that CE is endemic and a public health hazard in the study areas and the potential could be transmission to humans.

Almost all (96.7%) of livestock slaughtered were old animals beyond 3 years. This is because most livestock communities keep indigenous zebu and Ankole cattle which achieve slaughter weight at 3 and more years and hence longer exposure to *E. granulosus* eggs over time. Older animals above 3 years were more affected than younger ones. This was in agreement with the study by Terefe *et al*. (2012) in Ethiopia. There was very highly significant difference in CE prevalence between sexes, with males being highly infected. Although the specific reason for this is not clear, it could be postulated that males are heavier than females hence more male cattle appear in abattoirs for slaughter and hence more preferred for purchase by the cattle traders (butchers) than females. The higher prevalence of CE in older livestock >3 years could be due to cumulative exposure of older animals to CE infection over time. This was in agreement with suggestions by Torgerson *et al.* (1998); Larrieu *et al*. (2001) and Kebede *et al.* (2009*a*). Lung was the most affected organ in all livestock species: sheep, goats and cattle (Tables 4 and 5). This was in agreement with what was observed in Kenya (Kere *et al*., 2019), in Ethiopia (Getaw *et al*., 2010; (Negash *et al.*, 2013) and in China (Meng *et al*., 2014). In contrary, liver was the most affected organ in humans in the study area using ultrasound screening (Othieno *et al*., 2016). Furthermore, liver was the most livestock affected. This was found to be true in Kenya (Addy *et al*., 2012; Odongo *et al*., 2018; Nungari *et al*., 2019).

The lungs and livers were the most CE-infected. This is because they are the first organs rich in capillaries to be met by migrating *Echinococcus* oncospheres hence easily accessing the portal vein *via* the hepatic and pulmonary blood vessel system prior to any other peripheral organ. Also, the high infection rates in the lungs were probably due to their softer consistency thereby allowing easier development of the cysts with advancement in age (Himonas *et al.*, 1987). However, a majority of lung CE cysts were small sized. Liver and mixed organ infections had medium and large cysts. Similarly, the more the multi-organ infected the more the number of cysts recovered per organ (Table 7). For lung and liver 1.4 cysts were recovered per organ, 3.2 cysts were recovered per mixed lung and liver infection and 5 cysts per mixed lung, liver and spleen infection. This could mean the CE infection causing CE multi-organ infection was more aggressive due to different genotypes of infecting *E. granulosus* strains, host immunity, site, size of the cysts and geographical location, host, site, size and type of cyst may have different rates of fertility (Ibrahim, 2010). A study by Chamai *et al*. (2016) in Moroto found majorly *E. granulosus* sensu stricto – sheep (G3) strain and *E. canadensis* (G6/7) strains in slaughtered livestock and could partly explain this scenario. No fertile cysts were recovered from liver cysts of sheep, zebu cattle and goats. Most CE cysts recovered in the lungs and liver of goats, sheep and zebu cattle were calcified. This may suggest that goats, sheep and zebu cattle are slaughtered when they are older and by then most developed cysts had calcified. Ankole cattle had 33.9 and 4% fertile cysts in the liver and lungs respectively; sheep 1.7% and zebu cattle 6.4% had fertile cysts only in the liver, while goats had 4.5% fertile cysts in the lungs only. Fertile cysts pose greater risk for CE transmission when fed to dogs. This showed that sheep, goats and zebu cattle may potentially play a less significant role as intermediate hosts of CE in their localities as compared to Ankole cattle. *Cysticercus tenuicollis* is a tapeworm which has the same lifecycle as *Echinococcus* worms, sharing the same canids, especially dogs, as their final hosts, and herbivores as their intermediate hosts (Murell, 2005). Almost all sheep and goats which had CE infection also had *C. tenuicollis* infection in their mesenteries. There was high prevalence of *C. tenuicollis* in all districts of this study. This was because dogs had access to pastures that were being grazed by goats and sheep and hence can contaminate pastures with their fecal matter. The overall prevalence of 35.4 and 23.3% observed in goats and sheep respectively in this study was comparable to what has been observed among goats and sheep in Pairaba, Brazil of 39% in goats and 17.4% in sheep (de Morais et al., 2017) and in Ceara of 26.2% in goats and 35% in sheep (Soares *et al*., 2012) and in Uttar Pradesh, India of 27.3% in goats and 37.2% in sheep (Pathak and Gaur, 1982); in Soroti, Uganda of 42.5% in sheep and 33.3% in goats (Nyero *et al*., 2014) and in Nile Delta, Egypt of 21% in sheep (Abbas *et al*., 2021). More high prevalence rates have been observed among goats (53%) and sheep (79%) in eastern Ethiopia (Sissay *et al.*, 2008). Much lower prevalence had been observed in north India of 4.8% in goats and 2.2% in sheep (Singh *et al*., 2015), northeast Tunisia of 8.9% in goats and 2.2% in sheep (Khaled *et al*., 2020) and northwest Iran of 8.9% in goats and 2.8% in sheep (Mirzaei and Rezaei, 2015). *Cysticercus tenuicollis* though not zoonotic does not cause serious health effects in goats and sheep but have been found to lower their productivity (Soares *et al*., 2012; de Morais et al., 2017; Abbas *et al*., 2021). Therefore dogs should be dewormed to get rid of adult cestode worms which shed their eggs in dogs’ feces thereby contaminating pasture grazed by livestock.

In the present study, it has been observed that CE is endemic in pastoral and agro-pastoral areas in Uganda; higher prevalence was reported in sheep, goats and zebu cattle in Moroto compared to Kumi, Luwero and Nakasongola districts. Overall, the lungs were more affected than the liver. Higher cyst fertility rates in the liver of Ankole cattle, zebu cattle and sheep and lungs of goats suggest Ankole and zebu cattle, sheep and goats play an important role in the epidemiology of CE zoonosis and offer a great potential risk of transmission to humans.

*Cysticercus tenuicollis*, a similar type of tapeworm to *Echinococcus* spp. causing CE, was more prevalent in goats in all the study areas while sheep prevalence was observed only in Moroto and Kumi districts. This prevalence (*C. tenuicollis* and *E. granulosus* cysts) in food animals raises the possibility of dog infection from condemned infected organs, hence food and water contamination by abundant stray dogs’ infected fecal matter. This is responsible for disease transmission onto livestock and humans and therefore posing a serious public health risk. There is therefore an urgent need to create public health awareness among the communities about the need for proper and safe infected organ disposal, hence the epidemiology of both *C. tenuicollis* and *E. granulosus* in dogs, livestock and humans. Similar prevalence studies should also be undertaken in other areas of Uganda, and furthermore, assessment of financial losses due to CE, risk factor determination and CE molecular characterization in all livestock, dogs and humans. This will inform the relevant stakeholders in the animal and health sectors to enhance control strategies in livestock and the communities keeping them.

## Data Availability

Data supporting results are provided within the article.
